# Synthesis of SDS-Modified Pt/Ti_3_C_2_T_x_ Nanocomposite Catalysts and Electrochemical Performance for Ethanol Oxidation

**DOI:** 10.3390/nano11123174

**Published:** 2021-11-23

**Authors:** Beibei Yang, Tian Qin, Ziping Bao, Wenqian Lu, Jiayu Dong, Duan Bin, Hongbin Lu

**Affiliations:** 1 Department of Chemistry and Chemical Engineering, Nantong University, Nantong 226019, China; bbyang17@fudan.edu.cn (B.Y.); qt18036283600@163.com (T.Q.); baoziping9@163.com (Z.B.); Luwenqian1028@163.com (W.L.); 2 Institute of Materials Engineering, National Laboratory of Solid State Microstructures, College of Engineering and Applied Science, Nanjing University, Nanjing 210093, China; njudjy12345@163.com

**Keywords:** ethanol oxidation, sodium dodecyl sulfate (SDS) modified-Ti_3_C_2_T_x_, Pt nanoparticles, dispersion, electrocatalytic activity

## Abstract

It is well-known that platinum (Pt) is still the preferred material of anode catalyst in ethanol oxidation, however, the prohibitive high cost and CO poisoning of Pt metal impede the commercialization of fuel cells. Therefore, improving the utilization rate of catalysts and reduce the cost of catalyst become one of the most concerned focus in the construction of fuel cells. In this work, the Pt-based catalysts are synthesized by using different content of sodium dodecyl sulfate (SDS) modified-Ti_3_C_2_T_x_ support, and the dispersion regulation function of SDS modified-Ti_3_C_2_T_x_ supported on Pt nanoparticles is investigated. The structure, composition and morphology of different catalysts are characterized by X-ray diffraction (XRD), X-ray spectroscopy (EDX), transmission electron microscopy (TEM) and high-resolution TEM, respectively. It is found that the Pt nanoparticles in pure Ti_3_C_2_T_x_ surface are serious aggregated and show poor dispersion, whereas the Pt nanoparticles in SDS modified-Ti_3_C_2_T_x_ have a better dispersion. The electrochemical results revealed that SDS modified-Ti_3_C_2_T_x_ supported Pt nanoparticles has higher electrocatalytic activity and stability in both acidic and alkaline ethanol oxidation when the dosage of SDS increases to 100 mg. These findings indicate that the SDS-Ti_3_C_2_T_x_/Pt catalysts show a promising future of potential applications in fuel cells with modification of Ti_3_C_2_T_x_ support.

## 1. Introduction

With the continuous energy consumption and population growth, energy crisis has been a big problem of the world. In recent years, metal-air batteries (Li, Na, Zn) exhibit higher theoretical specific energy than traditional lithium ion batterie [1,2], however state-of-art metal-air batteries still produce performance that are well below practical levels. In contrast to the metal-air batteries, liquid fuel cell can be considered as a decent compromise between LIBs and metal-air batteries. Direct ethanol fuel cell (DEFC) is a liquid fuel cell with the advantages of high energy density, low environmental pollution, simple equipment structure, and easy preparation [3,4,5,6,7,8,9]. Platinum-based materials are generally used as the anode catalyst towards ethanol oxidation due to their excellent electrocatalytic performance. With the development of nanotechnology, researchers tend to design and synthesize nano-catalysts with controllable size and morphology, which could improve their catalytic activity. Liang et al. improved the stability of platinum-based catalysts by doping other atoms [10]. However, pure platinum catalyst is still expensive and can be easily poisoned by the intermediates from alcohol oxidation, limiting its commercial application in DEFC [11,12,13,14,15]. To resolve this problem, a more effective way is to explore a suitable catalyst carrier where the Pt nanoparticles can be uniformly dispersed and be well stabilized for a high catalytic efficiency.

Recently, a two-dimensional material composed of transition metal carbides, nitrides or carbonitrides, named as MXene have received attention for many electrochemical application owing to the high specific surface area, good electrical conductivity, rich surface functional group, and adjustable chemical structure [16,17,18,19,20]. Unlike graphene, MXene is difficult to obtained by mechanical peeling method because of its strong metal-bond between M and A in the precursor of MAX. In the existing research, MXene is mainly synthesized by etching the MAX under the fluoride hydrothermal conditions, where a strong-acidic HF solution or a mixed solution of LiF+HCl [21,22] is employed. By contrast, the LiF+HCl mixed etching solution is more feasible, avoiding the high risk of using high-concentration HF. Since MXene has large specific surface area, direct deposition of nanocomposite in MXene may lead to serious agglomeration, decreasing the catalytic activity of the catalysts. For example, Min et al. prepared Ti_3_C_2_T_x_ MXene nanosheet confined Pt nanoparticles to catalyze the photocatalytic hydrogen evolution reaction, whereas the Pt nanoparticles presented an unsatisfactory dispersion [22]. For this regard, surface modification of the MXene carrier by using different surfactants is an effective way to control the dispersion, the morphology, and the size of the nanoparticles. Li et al. studied the effects of cetyltrimethylammonium bromide (CTAB) surfactant for the MXene interlaminar functionalization and the dispersion of Se nanoparticles. As a result, the obtained Ti_3_C_2_@CTAB-Se composite displayed good electrochemical performance as the cathode electrode for aluminum ion batteries [23]. In addition, Wang et al. prepared the PDDA-functionalized MXene as carrier for the uniform dispersed Au nanoparticles through the adsorption of positively charged PDDA and MXene surface functional groups, which exhibited good catalytic performance in the electrochemical detection of sodium nitrite [24].

Among these investigated surfactants, the sodium dodecyl sulfate (SDS) was also found to improve the solubilization of carbon-based carries based on noncovalent interactions of SDS and carries surface. For instance, Li et al. designed a nonenzymatic H_2_O_2_ sensor based on Pt nanoparticles supported on SDS-modified multiwall carbon nanotube (PtNPs/SDS-MWCNTs) [25]. The SDS molecules were adsorbed on the surface of CNT, which could be regarded as protective agent for the deposition of Pt NPs. Considering previous versatility modulation of MXene and the surface function of SDS, we sought to employ SDS as the surfactant to systematically study the size and dispersion of Pt nanoparticles in the Ti_3_C_2_T_x_. The catalytic activity and stability of the SDS-Ti_3_C_2_T_x_/Pt electrocatalyst in the acidic and the alkaline ethanol solutions were systematically investigated. It is found that the electrocatalytic activity of the Pt-loaded Ti_3_C_2_T_x_ catalyst in ethanol oxidation is greatly improved by the functional modification of Ti_3_C_2_T_x_, which is also beneficial to catalyze the oxidation of the CO_ads_ intermediate product on the electrode surface.

## 2. Materials and Methods

### 2.1. Materials and Instruments

The materials include titanium aluminum carbide (Ti_3_AlC_2_) (Analytical grade, Macleans Reagent Co. Ltd., Shanghai, China), Lithium fluoride (LiF) (Analytical grade, Aladdin Reagent Co. Ltd., Shanghai, China), Chloroplatinic acid (H_2_PtCl_6_) (Aladdin Reagent Co. Ltd., Shanghai, China), borohydride Sodium (NaBH_4_) (Shanghai Lingfeng Chemical Reagent Co. Ltd., Shanghai, China), absolute ethanol (C_2_H_5_OH) (Shanghai Zhenxing Chemical Co. Ltd., Shanghai, China), and sodium dodecyl sulfate (SDS) (Analytical Pure, Aladdin Reagent Co. Ltd., Shanghai, China). Deionized (DI) water was used through the whole experiment.

HWCL-1 constant temperature stirrer (Zhengzhou Great Wall Technology Industry and Trade Co., Ltd., Zhengzhou, China), SB-5200D ultrasonic cleaning machine (Ningbo Xinzhi Biological Technology Co. Ltd., Ningbo, China), TGL-16 high-speed centrifuge (Shandong Baiou Co. Ltd., Nantong, China), transmission electron microscope (Thermo Fisher Talos F200x, Waltham, MA, USA), X-ray powder diffractometer (Bruck D8 Advance, Germany).

### 2.2. Synthesis of Ti_3_C_2_T_x_

Ti_3_C_2_T_x_ was prepared by etching method in the HCl+LiF mixed solution. Firstly, 1.6 g LiF was added to 20 mL 9 mol L^−1^ HCl, stir at 35 °C for half an hour, so that LiF was completely dissolved in HCl to obtain a transparent etching solution, and then Ti_3_AlC_2_ was slowly added to the HCl+LiF etching solution under stirring. In order to avoid violent exothermic reaction, stirring was kept for 24 h. The precipitated product was washed with ethanol and deionized water until the pH value of the supernatant was close to neutral. Finally, the product was placed in a vacuum oven to dry for 12 h, and the resultant was Ti_3_C_2_T_x_ powder.

### 2.3. Preparation of Ti_3_C_2_T_x_-Pt Composites Modified with Different Content of SDS

First, 1.0 g H_2_PtCl_6_ was dissolved in 250 mL of distilled water to form a solution with a concentration of 7.723 mmol L^−1^. 40 mg Ti_3_C_2_T_x_ powder prepared in the previous step was added to 40 mL deionized water, and ultrasonicated for 2 h. The SDS was employed as the surfactant to prevent the produced Pt nanoparticles to aggregation. In order to study the effect of SDS amounts on the electrocatalytic performance, different contents of SDS were added into the Ti_3_C_2_T_x_ solution and ultrasonicated for another 30 min. We set up four sets of samples, where 0 mg, 20 mg, 50 mg, and 100 mg of SDS were added into the Ti_3_C_2_T_x_ solution and the subsequent operations were the same as above. After then, 6.5 mL H_2_PtCl_6_ (7.723 M) solution was added the above solution under a magnetic stirrer for rapid stirring. 60 mg of NaBH_4_ solid was dissolved in 10 mL of deionized water, and the resulting solution was added to the above-mentioned H_2_PtCl_6_-Ti_3_C_2_T_x_ solution, and stirring was continued for 4 h. Then, the product was obtained by centrifugation, and washed twice with deionized water and ethanol successively. Finally, the product was put into an oven to dry at 60 °C for 12 h.

### 2.4. Characterization

X-ray powder diffractometer (XRD), scanning electron microscopy (SEM) and transmission electron microscopy (TEM) were applied to characterize and analyze the morphology and structure of the prepared samples. The samples were carried on Fourier transform infrared spectroscopy (FTIR) with a Nicolet 6700 FTIR spectrometer instrument using a KBr pellet, and Raman spectrometer (LABRAM-1B) with a 514 nm laser source. The oxidation states of and samples were obtained by X-ray photoelectron spectroscopy (XPS, XSAM800 Ultra spectrometer). A three-electrode system was used to conduct electrochemical tests by the CHI660E electrochemical workstation. The reference electrode was a saturated calomel electrode, the counter electrode was a platinum electrode, and the working electrode was a glassy carbon electrode coated with the prepared catalysts (working area is 0.07 cm^2^).The working electrode was prepared as follows: 5 mg of catalyst powder was dissolved in a mixed solution consisting of 850 μL of deionized water, 100 μL of isopropanol and 50 μL of Nafion, and then ultrasonicated for half an hour to form a uniformly dispersed catalyst solution. 10 μL of the catalyst solution was hanging dropped on the surface of the polished glassy carbon electrode by pipette and dried under infrared lamp. The commercial JM 20 wt% Pt/C catalyst was prepared by the same procedure.

## 3. Results and Discussion

### 3.1. Physical Characterization

**Figure 1:**
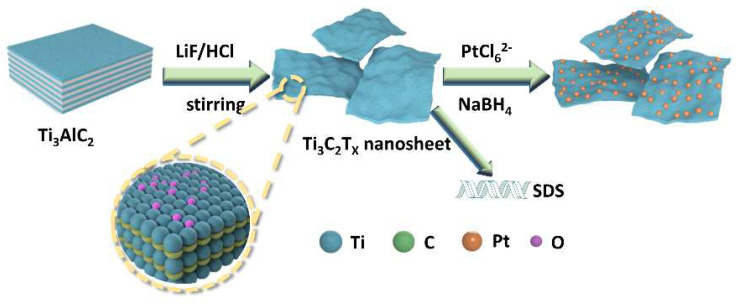
Schematic illustration of SDS modified-Ti_3_C_2_T_x_/-Pt nanocomposites.

**Figure 2:**
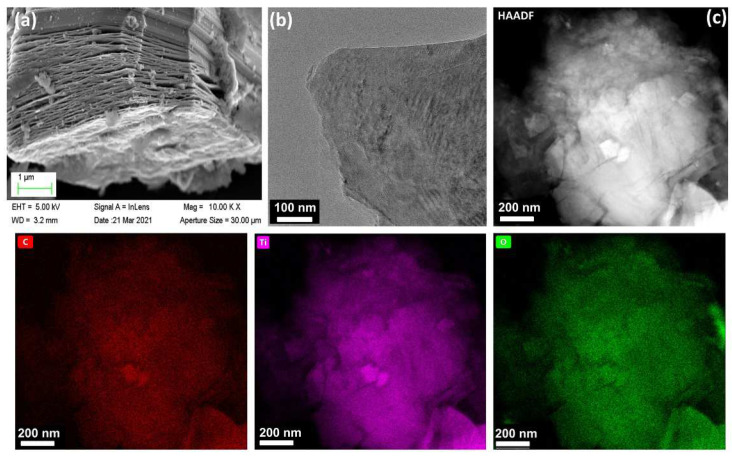
(**a**) SEM, (**b**) TEM and (**c**) HAADF-STEM images of Ti_3_C_2_T_x_ product from etching Ti_3_AlC_2_ precursor. EDX mappings of C, Ti and O.

**Figure 3:**
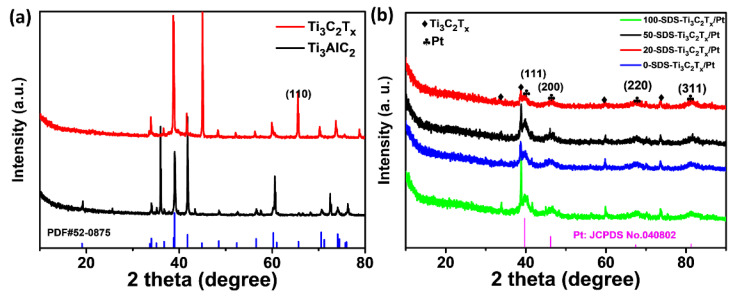
XRD patterns of (**a**) initial and etching Ti_3_AlC_2_, (**b**) XRD patterns of 0-SDS-Ti_3_C_2_T_x_/Pt, 20-SDS-Ti_3_C_2_T_x_/Pt, 50-SDS-Ti_3_C_2_T_x_/Pt, 100-SDS-Ti_3_C_2_T_x_/Pt nanocomposites.

The preparation process of the SDS-modified Ti_3_C_2_T_x_-Pt composite material was shown in Figure 1. First, the Ti_3_AlC_2_ powder was etched into a multilayer structure by LiF and HCl. Then, the multilayer Ti_3_C_2_T_x_ is modified with SDS and sonicated into SDS-modified Ti_3_C_2_T_x_ support, and the Pt nanoparticles is deposited on the SDS-modified Ti_3_C_2_T_x_ support by serving NaBH_4_ as reducing expanded graphite after the Al layer was selectively removed under etching. It can be found from the SEM (Figure 2a) that the obtained Ti_3_C_2_T_x_ exhibits an accordion layered structure, the TEM (Figure 2b) image and EDX mapping show a thin and semitransparent nanosheet with a uniform distribution of the Ti and C elements from Ti_3_C_2_T_x_. The structure of as-prepared SDS-Ti_3_C_2_T_x_/Pt composites were analyzed by X-ray powder analyzer and the results were shown in Figure 3. In Figure 3a, a series of strong and sharp characteristic diffraction peaks are observed in the initial T_i3_AlC_2_ powder, corresponding to the hexagonal crystal (JCPDS. No.520875). After etching, original characteristic peaks disappear, and new peaks associated with Ti_3_C_2_T_x_ were appears, indicating the successful synthesis of Ti_3_C_2_T_x_. Figure 3b shows the XRD spectra of the 0-SDS-Ti_3_C_2_T_x_/Pt, 20-SDS-Ti_3_C_2_T_x_/Pt, 50-SDS-Ti_3_C_2_T_x_ /Pt, and 100-SDS-Ti_3_C_2_T_x_/Pt. Most of the nanocomposites show the Ti_3_C_2_T_x_ characteristic diffraction peaks at 2θ = 33.9°, 38.8°, 60.1° and 73.6°. The characteristic diffraction peaks of Pt appear at the positions of 39.8, 46.4, 67.6, and 81.2°, corresponding to the (111), (200), (220), and (311) crystal planes of the cubic structure (JCPDS. No. 040802), respectively [26,27]. In addition, SDS-modified Ti_3_C_2_T_x_-Pt composite nearly resembles the pure Ti_3_C_2_T_x_ in terms of the position of Raman bands, only the vibration mode at 482 cm^−2^ is gradually disappeared when the SDS increased to 50 mg (Figure S1). The result revealed the A_1g_ out-of-plane vibration modes at 154 and 619 cm^−2^ for C and Ti atoms, and the E_g_ in-plane vibration modes at 207 and 333 cm^−2^ for C, Ti and surficial groups [28]. FT-IR spectra (Figure S2) manifests the rich surface chemistry of MXene structure by the adsorption peaks corresponding to -OH asymmetric stretch (3446 cm^−1^), -OH asymmetric vibration (2916 cm^−1^), -OH bending vibration (1627 cm^−1^), Ti-C-O (1107 cm^−1^), which is consisted with the previous work [29].

**Figure 4:**
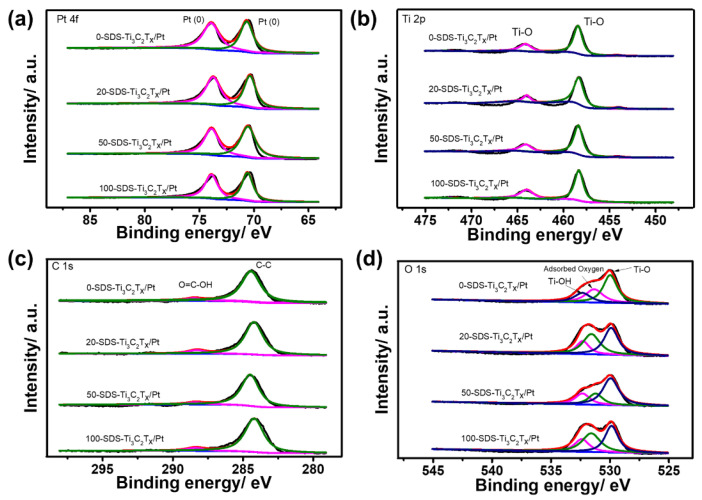
High-resolution XPS spectrum of Pt 4f (**a**), Ti 2p (**b**), C 1s (**c**) and O 1s (**d**) for 0-SDS-Ti_3_C_2_T_x_/Pt, 20-SDS-Ti_3_C_2_T_x_/Pt, 50-SDS-Ti_3_C_2_T_x_/Pt and 100-SDS-Ti_3_C_2_T_x_/Pt nanocomposites.

**Figure 5:**
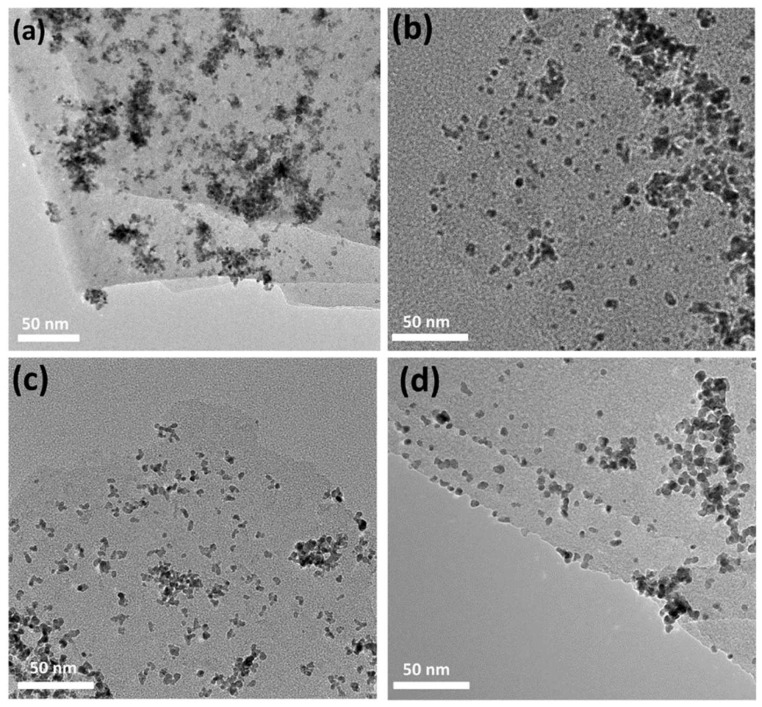
TEM images of (**a**) 0-SDS -Ti_3_C_2_T_x_/Pt, (**b**) 20-SDS-Ti_3_C_2_T_x_/Pt, (**c**) 50-SDS-Ti_3_C_2_T_x_/Pt, (**d**) 100-SDS-Ti_3_C_2_T_x_/Pt catalysts.

XPS measurements were further used to analyze the composition and surface oxidation state of the as-prepared catalysts. Figure 4 shows the high-resolution XPS spectra of 0-SDS-Ti_3_C_2_T_x_/Pt, 20-SDS-Ti_3_C_2_T_x_/Pt, 50-SDS-Ti_3_C_2_T_x_/Pt and 100-SDS-Ti_3_C_2_T_x_/Pt catalysts. As observed, the survey spectra evidenced the existence of C, Ti, Pt, O elements and the similar position for the four catalysts. As shown in Figure 4a, the SDS-Ti_3_C_2_T_x_/Pt displays two prominent peaks at binding energies of 70.7 and 73.9 eV, corresponding to the Pt 4f_7/2_ and Pt 4f_5/2_ of metallic Pt [30]. The Ti 2p spectrum could be deconvoluted into two peaks at 464.1 and 458.3 eV, which can be ascribed to the Ti-O (2p_1/2_) and Ti-O (2p_3/2_) group, respectively (Figure 4b). For the XPS spectra of C 1s, the two peaks at 284.4 and 285.5 eV seen in Figure 4c correspond to the C-C and O=C-OH group, respectively. The peaks with binding energies of 529.9, 531.4 and 532.3 eV can be assigned to the Ti–O, chemisorbed or physically absorbed oxygen and Ti–OH species (Figure 4d), indicating the presence of abundant surface oxygenated groups on the surface of Ti_3_C_2_T_x_ [20]. In order to determine the effect of the surfactant SDS content on the morphology and dispersibility of Pt nanoparticles, the microstructure of the nanocomposite catalyst was observed in Figure 5. It can be seen from Figure 5a that Pt nanoparticles are severely agglomerated on the Ti_3_C_2_T_x_ carrier without SDS modification. However, when the Ti_3_C_2_T_x_ is modified by SDS, Pt nanoparticles are uniformly dispersed and exhibit a smaller particle size on the Ti_3_C_2_T_x_ surface, as shown in Figure 5b–d. In addition, the more content of the SDS surfactant is added, the better dispersion of Pt nanoparticles could be achieved. Generally speaking, nanoparticles with a smaller particle size and better dispersion provide more active sites for electrochemical reactions, thereby increasing the catalytic activity [31].

**Figure 6:**
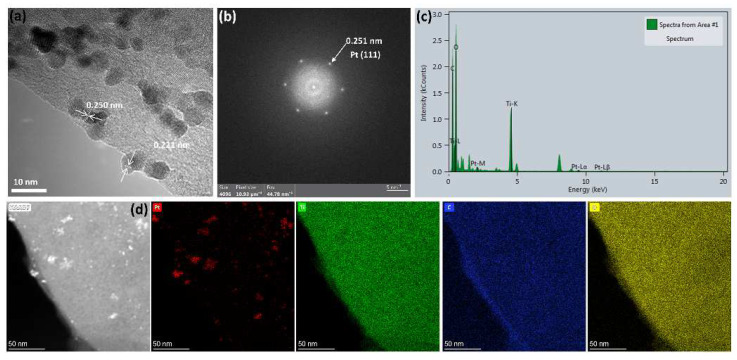
(**a**) HRTEM, (**b**) SAED pattern and (**c**) EDX element analysis and (**d**) mappings of 100-SDS-Ti_3_C_2_T_x_/Pt nanocomposite.

Figure 6a–c displays the HRTEM, SAED, and EDX patterns of the 100-SDS-Ti_3_C_2_T_x_/Pt nanocomposite, and the HRTEM confirms the crystalline phase structure of Pt. It can be estimated from Figure 6a, that the interplanar spacing of Pt nanoparticles is 0.221 and 0.250 nm, corresponding to the Pt (111) and the Pt (200) crystal-plane of face-centered cubic crystal. From the SAED in Figure 6b, the interplanar spacing of 0.251 nm is observed, which is consistent with the Pt (200) crystal-plane in Figure 6a. In addition, the EDX spectrum in Figure 6c also confirms the presence of Pt in the nanocomposite catalyst. Among them, the peaks of C and Ti are mainly derived from Ti_3_C_2_T_x_, and the signal of the O peak is attributed to the oxygen-containing functional groups of Ti_3_C_2_T_x_ and the surface oxygen of copper mesh. Figure 6d shows the EDX mapping, in which a uniform distribution of the Ti, C and O elements from Ti_3_C_2_T_x_ nanosheets is observed, while the Pt element is highly dispersed on the Ti_3_C_2_T_x_ nanosheets in the form of small Pt nanoparticles.

### 3.2. Electrochemical Characterization

**Figure 7:**
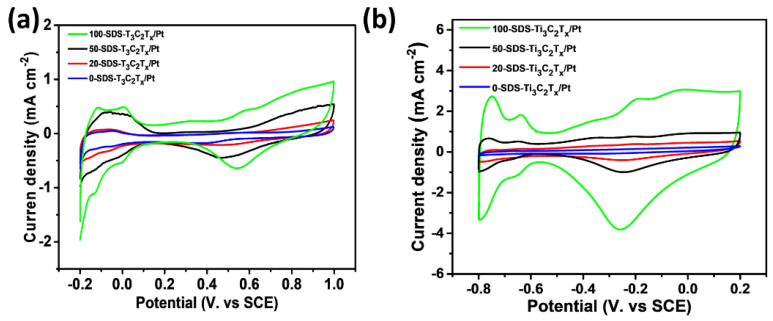
CV curves obtained from (**a**) 0.5 mol L^−1^ H_2_SO_4_ and (**b**) 1 mol L^−1^ KOH for 0-SDS-Ti_3_C_2_T_x_/Pt, 20-SDS-Ti_3_C_2_T_x_/Pt, 50-SDS-Ti_3_C_2_T_x_/Pt and 100-SDS-Ti_3_C_2_T_x_/Pt nanocomposites, scan rate of 50 mV s^−1^.

In order to study the electrocatalytic activity of the catalyst, cyclic voltammetry (CV) curves were used to investigate the electrocatalytic activity of different catalysts in the acidic ethanol and the alkaline ethanol. The CV curves of 0-SDS-Ti_3_C_2_T_x_/Pt, 20-SDS-Ti_3_C_2_T_x_/Pt, 50-SDS-Ti_3_C_2_T_x_/Pt and 100-SDS-Ti_3_C_2_T_x_/Pt catalysts in 0.5 mol L^−1^ H_2_SO_4_ solution were measured. As shown in Figure 7a, the cathode and anode peaks with the potential range of −0.2 V to 0.1 V correspond to the absorption peak and desorption peak of monolayer hydrogen under acidic conditions, respectively. The electrochemically active area (ECSA) of the electrode can be estimated from the peak area of the hydrogen absorption and desorption [32,33]. ECSA = Q_h_/(0.21 × m), where Q_h_ represents the amount of electricity during hydrogen desorption, and m is the content of Pt on the electrode. According to this formula, the ECSA of 100-SDS-Ti_3_C_2_T_x_/Pt, 50-SDS-Ti_3_C_2_T_x_/Pt, 20-SDS-Ti_3_C_2_T_x_/Pt, 0-SDS-Ti_3_C_2_T_x_/Pt nanocomposite catalyst is 9.02, 5.67, 2.60, 1.80 m^2^ g^−1^, respectively. The larger ECSA is, the higher catalytic activity is achieved in the acidic media. Figure 7b shows the CV curve of 0-SDS-Ti_3_C_2_T_x_/Pt, 20-SDS-Ti_3_C_2_T_x_/Pt, 50-SDS-Ti_3_C_2_T_x_/Pt, and 100-SDS-Ti_3_C_2_T_x_/Pt nanocomposites in 1 mol L^−1^ KOH solution. The sweep potential range is −1.0 V~0.2 V. As shown in this figure, during the reverse scanning process, all catalysts have obvious redox peaks at −0.25 V. Among these catalysts, the 100-SDS-Ti_3_C_2_Tx /Pt catalyst has the largest peak current density, indicating the highest electrocatalytic activity. As mentioned above, the ECSA value of 100-SDS-Ti_3_C_2_T_x_/Pt is obvious higher that of 50-SDS-Ti_3_C_2_T_x_/Pt, 20-SDS-Ti_3_C_2_T_x_/Pt and 0-SDS-Ti_3_C_2_T_x_/Pt electrode, rendering the highest electrochemical activity among these catalysts. This is attributed to the better dispersion of catalyst nanoparticles on the surface of Ti_3_C_2_T_x_ with more SDS surfactant, which increases the more active sites for electrochemical reactions.

**Figure 8:**
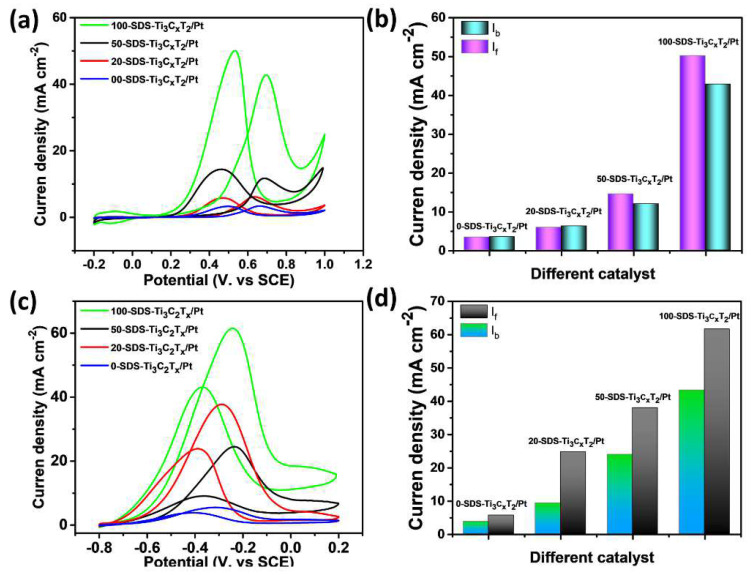
CV curves obtained in (**a**,**b**) 0.5 mol L^−1^ H_2_SO_4_ +1 mol L^−1^ C_2_H_5_OH and (**c**,**d**) 1 mol L^−1^ KOH+1 mol L^−1^ C_2_H_5_OH for 0-SDS-Ti_3_C_2_T_x_/Pt, 20-SDS-Ti_3_C_2_T_x_/Pt, 50-SDS-Ti_3_C_2_T_x_/Pt and 100-SDS-Ti_3_C_2_T_x_/Pt nanocompsites, scan rate of 50 mV s^−1^.

Figure 8a shows the CV curves of the four nanocatalysts in 0.5 mol L^−1^ H_2_SO_4_+1 mol L^−1^ C_2_H_5_OH solution. There are two obvious oxidation peaks observed at 0.65 V and 0.46 V, which is assigned to the positive oxidation peak of ethanol and the oxidation peak of the toxic intermediate product during the negative scan. Generally, a higher the peak current of ethanol during forward scanning means a better catalytic performance. The current density of the 100-SDS-Ti_3_C_2_T_x_/Pt nanocatalyst reaches 50.3 mA cm^−2^, which is much higher than 0-SDS-Ti_3_C_2_T_x_/Pt, 20-SDS-Ti_3_C_2_T_x_/Pt, and 50-SDS-Ti_3_C_2_T_x_/Pt, indicating its largest electrocatalytic activity. The ratio (j_f_/j_b_) between the current density of the positive-scan oxidation peak (j_f_) and the peak current density (j_b_) of the anti-scan oxidation peak (j_f_/j_b_) is employed to study the anti-toxicity of the catalyst during the ethanol oxidation process. The higher ratio (j_f_/j_b_) is, the easier oxidation reaction of ethanol is, and the nanocomposite can adsorb ethanol molecules more efficiently [34,35]. As shown in Figure 8b, it can be clearly found the j_f_/j_b_ value of 100-SDS-Ti_3_C_2_T_x_/Pt catalyst is 1.21, which is higher than 50-SDS-Ti_3_C_2_T_x_/Pt (1.17), 20-SDS- Ti_3_C_2_T_x_/Pt (0.94), and 0-SDS-Ti_3_C_2_T_x_/Pt (0.97), further demonstrating its high electrocatalytic activity and anti-poisoning ability for ethanol oxidation in acidic media. Owing to the superior electrocatalytic activity of 100-SDS-Ti_3_C_2_T_x_/Pt over other catalyst, we also compared the electrochemical performance of 100-SDS-Ti_3_C_2_T_x_/Pt and commercial Pt/C catalysts, and the results were shown in Figure S3. In acid media, the current density and the j_f_/j_b_ value of 100-SDS-Ti_3_C_2_T_x_/Pt is obviously higher than that of (46.4 mA cm^−2^, 0.94). Furthermore, the CA after 6000 s (Figure S3b) also showed that, the current decay of 100-SDS-Ti_3_C_2_T_x_/Pt is much slower than that of Pt/C catalyst. Taken together, these results indicated that the 100-SDS-Ti_3_C_2_T_x_/Pt have remarkable catalytic and long-term stability for ethanol oxidation in acid solution. The electrocatalytic activity of 100-SDS-Ti_3_C_2_T_x_/Pt, 50-SDS-Ti_3_C_2_T_x_/Pt, 20-SDS-Ti_3_C_2_T_x_/Pt, and 0-SDS-Ti_3_C_2_T_x_/Pt catalysts in alkaline media is shown in Figure 8c. Similarly, oxidation peaks appear during the positive scanning process and the reverse scanning process. The oxidation peak during the positive scanning process is the direct oxidation peak of ethanol, and the oxidation peak during the negative scanning process is the re-oxidation of the intermediate product. In the mixture solution of 1 mol L^−1^ KOH and 1 mol L^−1^ C_2_H_5_OH, the direct oxidation peak current densities of ethanol are 61.8, 37.8, 24.6, and 5.5 cm^−2^ with the electrode of 100-SDS-Ti_3_C_2_T_x_/Pt, 50-SDS-Ti_3_C_2_T_x_/Pt, 20-SDS-Ti_3_C_2_T_x_/Pt, and 0-SDS-Ti_3_C_2_T_x_/Pt, respectively. Obviously, the 100-SDS-Ti_3_C_2_T_x_/Pt catalyst obviously has the largest current density, indicating the highest electrocatalytic activity in the alkaline medium. The ratios of j_f_/j_b_ for 100-SDS-Ti_3_C_2_T_x_/Pt, 50-SDS-Ti_3_C_2_T_x_/Pt, 20-SDS-Ti_3_C_2_T_x_/Pt, and 0-SDS-Ti_3_C_2_T_x_ /Pt are 1.40, 1.58, 2.66 and 1.50, respectively, as shown in Figure 8d. The 100-SDS-Ti_3_C_2_T_x_/Pt catalyst has a lower j_f_/j_b_ value, which indicates that the catalyst with the highest catalytic activity could also bring in more intermediate products in the secondary oxidation process. Figure S4 shows the electrocatalytic activity of the 100-SDS-Ti_3_C_2_T_x_/Pt and commercial Pt/C catalysts for ethanol oxidation in alkaline solution. The forward anodic peak value of 100-SDS-Ti_3_C_2_T_x_/Pt is slightly higher than 48.0 cm^−2^ for commercial Pt/C catalyst (Figure 4a). After 6000 s of CA (Figure 4b), the residue current of 100-SDS-Ti_3_C_2_T_x_/Pt is much higher that of Pt-C, indicating a superior stability and catalytic activity towards ethanol oxidation in alkaline solution. In addition, it is observed that the electrocatalytic activity of the 100-SDS-Ti_3_C_2_T_x_/Pt nanocatalyst in the alkaline medium is significantly stronger than that of in the acidic medium. This is because ethanol oxidation involves multiple steps in the dehydrogenation process. The platinum on the catalyst surface is easily oxidized by the surrounding water molecules to form the intermediate species, Pt+H_2_O→Pt-OH_ads_+H^+^+e^−^. In the acidic solutions, due to the low content of oxygen-containing substances OH_ads_, the ability of Pt to catalyze the oxidative dehydrogenation of ethanol is relatively poor [36]. However, the alkaline solution contains abundant OH groups, which makes intermediate products prone to dehydrogenation reaction, significantly improving the electrocatalytic efficiency of ethanol oxidation. The reaction is as follows: CH_3_CH_2_OH+2OH^−^→CH_3_COOH+2H_2_O+2e^−^. The process of alkaline oxidation can be deduced that ethanol molecules are first adsorbed on the catalyst and interact with the OH_ads_ in the solution, then gradually dehydrogenate to form CH_3_CO^−^ or CH_3_COO^−^, and finally the C-C bonds are break to form CO_2_.

**Figure 9:**
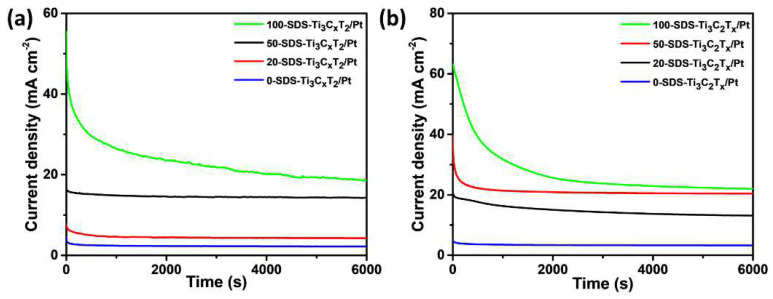
I-t curves in (**a**) 0.5 mol L^−1^ H_2_SO_4_+1 mol L^−1^ C_2_H_5_OH and (**b**) 1 mol L^−1^ KOH+1 mol L^−1^ C_2_H_5_OH solution for 0-SDS-Ti_3_C_2_T_x_/Pt, 20-SDS-Ti_3_C_2_T_x_/Pt, 50-SDS-Ti_3_C_2_T_x_/Pt and 100-SDS Ti_3_C_2_T_x_/Pt catalysts after 6000 s.

In order to compare the stability of the 0-SDS-Ti_3_C_2_T_x_/Pt, 20-SDS-Ti_3_C_2_T_x_/Pt, 50-SDS-Ti_3_C_2_T_x_/Pt and 100-SDS Ti_3_C_2_T_x_/Pt catalysts in the electrocatalytic process, the chronoamperometry was employed to analyze the performance of catalysts in the mixed solutions of 0.5 mol L^−1^ H_2_SO_4_+1 mol L^−1^ C_2_H_5_OH and 1 mol L^−1^ KOH+ 1 mol L^−1^ C_2_H_5_OH. Generally, the attenuation of the oxidation current density means the decrease of catalyst activity and the catalyst poisoning, which results from the adsorption of the CO intermediate product from the oxidation of ethanol on the surface of Pt [37,38]. As shown in Figure 9a, the current density of all catalysts decreases significantly in the beginning and then gradually stabilized. The results show that the 100-SDS-Ti_3_C_2_T_x_/Pt nanocomposite still has the highest current density after 6000 s chronoamperometry test, indicating its better anti-poisoning ability and stability. Besides, in 1 mol L^−1^ KOH+1 mol L^−1^ C_2_H_5_OH solution, the final steady-state current density of the 100-SDS-Ti_3_C_2_T_x_/Pt catalyst is also highest than others (Figure 9b). These results confirm that 100-SDS-Ti_3_C_2_T_x_/Pt has the best anti-poisoning ability and stability during the electrocatalytic oxidation of ethanol, no matter in the acid or the alkaline media, which is consistent with the previous CV results. Moreover, we measured the repeatability of electrocatalytic activity for the 100-SDS-Ti_3_C_2_T_x_/Pt catalyst, and the results were shown in Figure S5. After the catalyst has been put aside for one month, the current density of 100-SDS-Ti_3_C_2_T_x_/Pt still kept 92.8% and 87.2% of its initial current density for acid (Figure S5a) and alkaline solution (Figure S5b). Although the higher catalytic activity of alkaline solution that acid solution, the100-SDS-Ti_3_C_2_T_x_/Pt catalyst displayed a better repeatability in acid solution.

**Figure 10:**
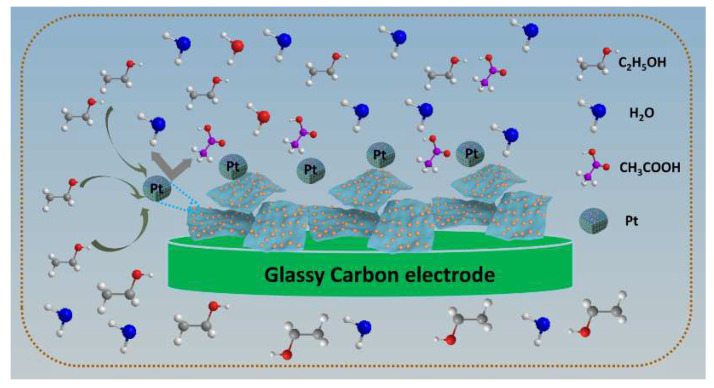
Schematic illustration the ethanol oxidation mechanism on the surface of SDS-Ti_3_C_2_T_x_/Pt electrodes in acidic and alkaline solution.

The generally accepted oxidation mechanism of SDS-Ti_3_C_2_T_x_/Pt catalysts is described in the Figure 10 and the following equation from the previous research [39,40]. The electrocatalytic oxidation mechanism of ethanol on Pt electrode involves parallel reaction and continuous reaction mechanism, the main product is CH_3_COOH or other products such as CH_3_CHO, CH_3_, CO_2_ and CO. Most specifically, ethanol is first adsorbed the surface of Pt and occurs the oxidation to CH_3_CHO_ads_ (Equations (1) and (2)). The produced poisonous intermediates Pt-(CH_3_CHO)_ads_ occupy most of the active sites to form catalyst deactivation. The Pt-(CH_3_CHO)_ads_ are oxidized to acetic acid by these active Pt-containing species and adsorbed Pt is meanwhile released, which is the rate-determining step in this process(Equation (3)).

Pt + C_2_H_5_OH→Pt-C_2_H_5_OH_ads_ (1)

Pt-C_2_H_5_OH_ads_ + 2OH^−^→Pt-(CH_3_CHO)_ads_ + 2H_2_O + 2e^−^ (2)

Pt-(CH_3_CHO)_ads_ + Pt-(OH)_ads_ + OH^−^→2Pt + CH_3_COOH + H_2_O + e^−^ (3)

In the ethanol oxidation process, there must be sufficient intermediate adsorption species and OH^−^ group on the catalyst surface. The 100-SDS-Ti_3_C_2_T_x_/Pt catalyst could provide more active sites over other catalysts, it is beneficial to dissociation and adsorption of ethanol molecular and display a good catalytic performance.

## 4. Conclusions

In this paper, a two-step synthesis method was used to synthesize the SDS-Ti_3_C_2_T_x_/Pt nanocomposite catalyst. The effect of the SDS content on the dispersion of Pt nanoparticles was studied and the electrocatalytic performance for ethanol oxidation of the SDS-Ti_3_C_2_T_x_/Pt nanocomposite catalyst in the acidic and in the alkaline media was also investigated. The SDS-Ti_3_C_2_T_x_ supported catalysts not only improve the catalytic performance of Pt catalyst towards EOR, but also considerably decrease the cost of noble catalyst. The electrocatalytic activity of the SDS-Ti_3_C_2_T_x_/Pt nanocomposite catalyst reaches the maximum when 100 mg SDS is added. The improved electrocatalytic activity and anti-poisoning ability of SDS-Ti_3_C_2_T_x_/Pt is attributed to the SDS modification for Ti_3_C_2_T_x_, which increases the dispersion and the active sites of Pt nanoparticles. This work provides important basics for the synthesis and application of Pt nanoparticle in catalysis.

## Data Availability

The data is available on reasonable request from the corresponding author.

## Supplementary Materials

The following are available online at https://www.mdpi.com/article/10.3390/nano11123174/s1, Figures S1–S5: Raman spectra, FT-IR spectra and CV curves.
