# Supplementary material for: Synthesis of SDS-Modified Pt/Ti3C2Tx Nanocomposite Catalysts and Electrochemical Performance for Ethanol Oxidation
Source: Nanomaterials (Basel). 2021 Nov 23;11(12):3174. doi: 10.3390/nano11123174 (PMC8703315; doi:10.3390/nano11123174)
Supplement: Supplementary file 1 [file nanomaterials-11-03174-s001.zip › nanomaterials-1451094-supplementary.pdf]

Synthesis of SDS-modified Pt/$Ti_3C_2T_x$ nanocomposite catalysts and electrochemical performance for ethanol oxidation

Beibei Yang [1], Tian Qin [1], Ziping Bao [1], Wenqian Lu [1], Jiayu Dong [2], Duan Bin [1,*] and Hongbin Lu [1,*]

1 Department of Chemistry and Chemical Engineering, Nantong University, Nantong, 226019, China
2 College of Engineering and Applied Science, Nanjing University, 210093, China
* Correspondence: dbin17@fudan.edu.cn; luhb@ntu.edu.cn)

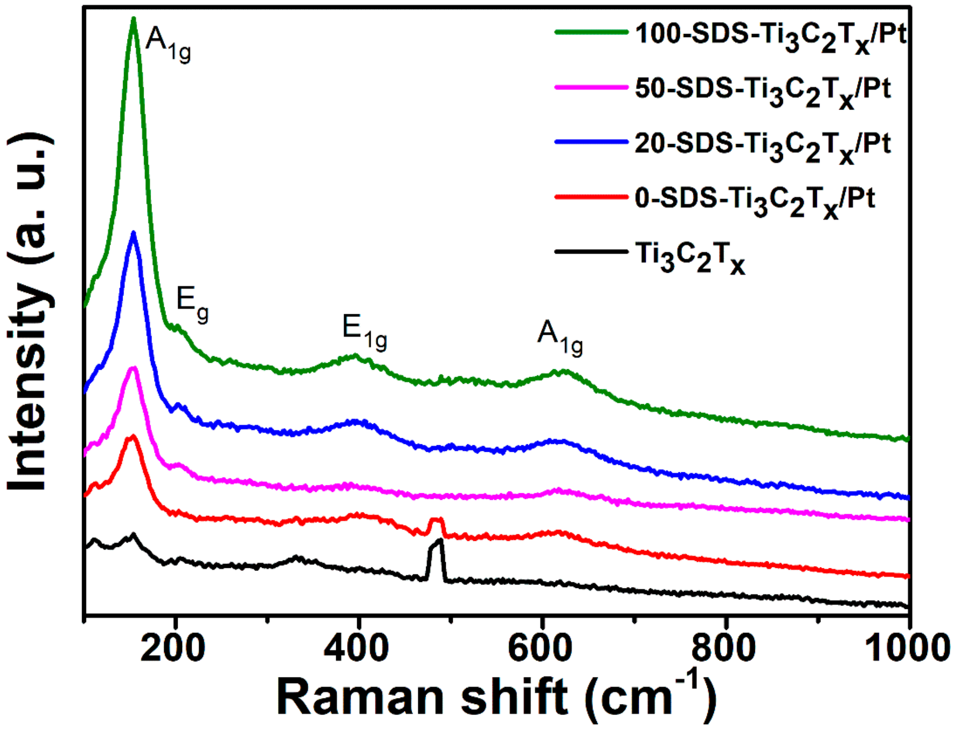

Figure S1. Raman spectra of 0-SDS-$Ti_3C_2T_x$/Pt, 20-SDS-$Ti_3C_2T_x$/Pt, 50-SDS-$Ti_3C_2T_x$/Pt and 100-SDS-$Ti_3C_2T_x$/Pt and pure $Ti_3C_2T_x$ samples.

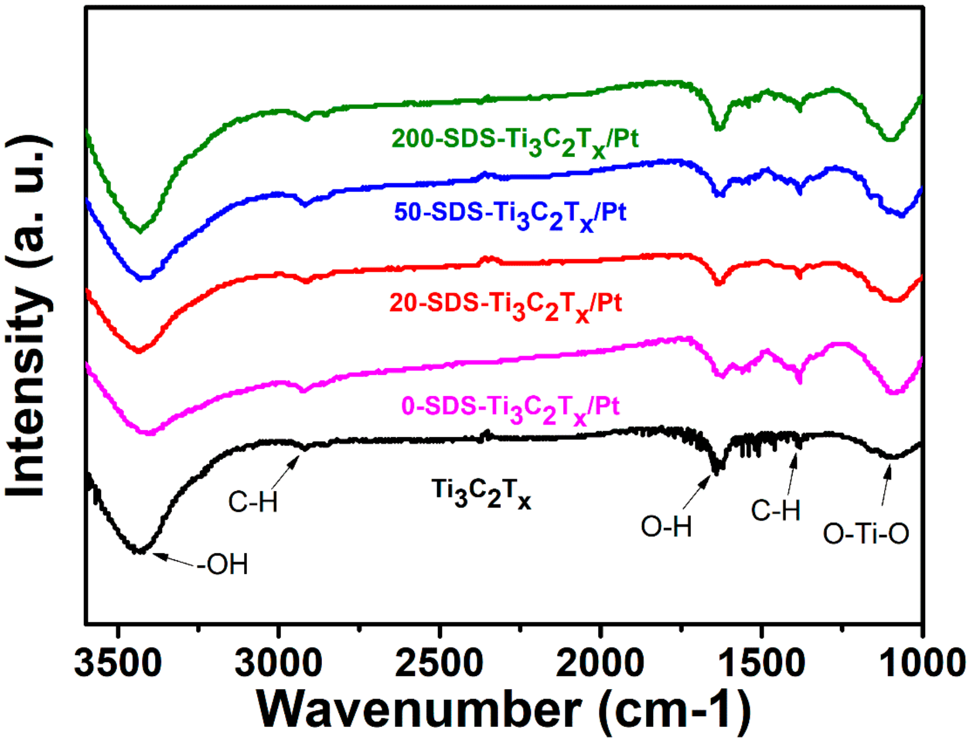

Figure S2. FT-IR spectra of 0-SDS-$Ti_3C_2T_x$/Pt, 20-SDS-$Ti_3C_2T_x$/Pt, 50-SDS-$Ti_3C_2T_x$/Pt and 100-SDS-$Ti_3C_2T_x$/Pt and pure $Ti_3C_2T_x$ samples.

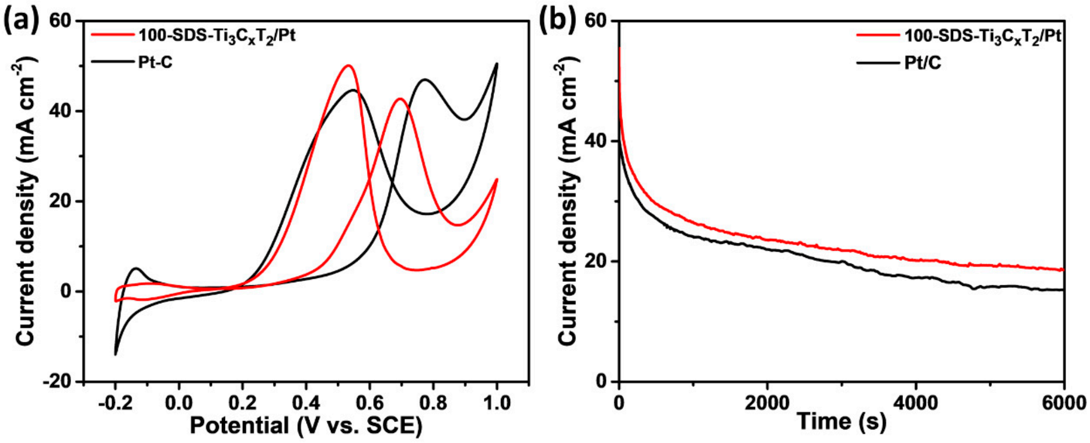

Figure S3. (a) CV curves and (b) I-t curves of Pt/C and 100-SDS-$Ti_3C_2T_x$/Pt catalysts in 0.5 mol L^{-1} H_2SO_4 +1 mol L^{-1} C_2H_5OH solution.

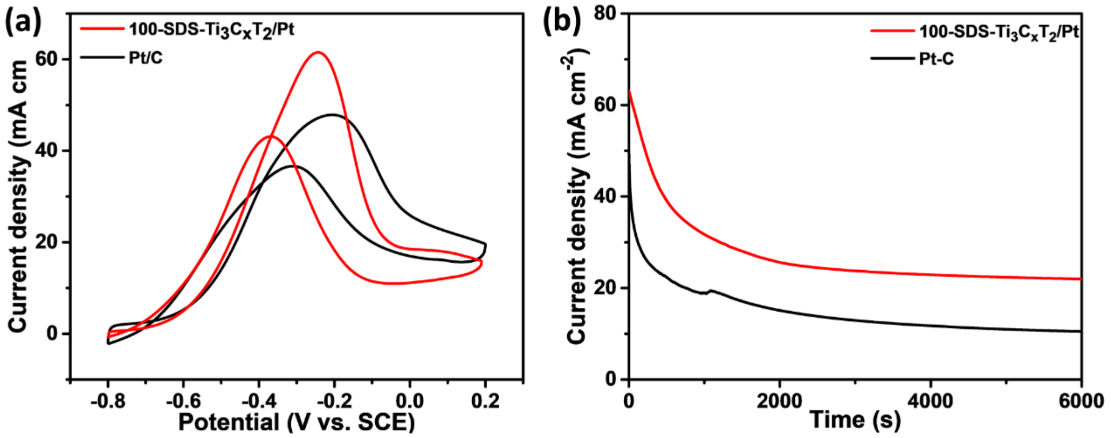

Figure S4. (a) CV curves and (b) I-t curves of Pt/C and 100-SDS-$Ti_3C_2T_x$/Pt catalysts in 1 mol L^{-1} KOH +1 mol L^{-1} C_2H_5OH solution.

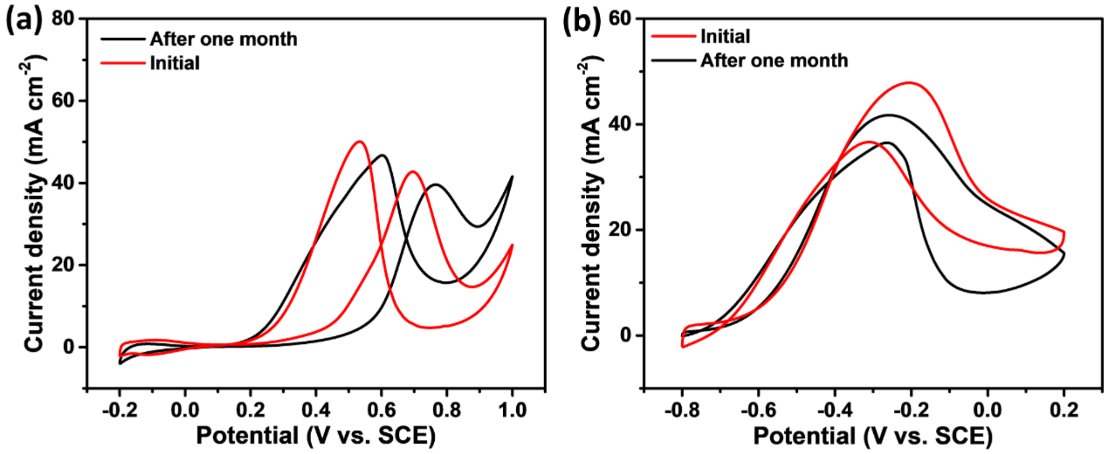

Figure S5. Initial CV result and CV result after one month for the 100-SDS-$Ti_3C_2T_x$/Pt in (a) 0.5 mol L^{-1} H_2SO_4 +1 mol L^{-1} C_2H_5OH solution and (b) 1 mol L^{-1} KOH +1 mol L^{-1} C_2H_5OH solution. .
